# Construction of a prognostic model for extensive-stage small cell lung cancer patients undergoing immune therapy in northernmost China and prediction of treatment efficacy based on response status at different time points

**DOI:** 10.1007/s00432-024-05767-6

**Published:** 2024-05-15

**Authors:** Junjie Dang, Gang Xu, Ge Guo, Huan Zhang, Lihua Shang

**Affiliations:** Department of Medical Oncology, Harbin Medical University Cancer Hospital, Harbin, 150000 Heilongjiang China

**Keywords:** Small cell lung cancer, Prognostic model, Immunotherapy, Long term survivors

## Abstract

**Background and purpose:**

Recently, the emergence of immune checkpoint inhibitors has significantly improved the survival of patients with extensive-stage small cell lung cancer. However, not all patients can benefit from immunotherapy; therefore, there is an urgent need for precise predictive markers to screen the population for the benefit of immunotherapy. However, single markers have limited predictive accuracy, so a comprehensive predictive model is needed to better enable precision immunotherapy. The aim of this study was to establish a prognostic model for immunotherapy in ES-SCLC patients using basic clinical characteristics and peripheral hematological indices of the patients, which would provide a strategy for the clinical realization of precision immunotherapy and improve the prognosis of small cell lung cancer patients.

**Methods:**

This research retrospectively collected data from ES-SCLC patients treated with PD-1/PD-L1 inhibitors between March 1, 2019, and October 31, 2022, at Harbin Medical University Cancer Hospital. The study data was randomly split into training and validation sets in a 7:3 ratio. Variables associated with patients’ overall survival were screened and modeled by univariate and multivariate Cox regression analyses. Models were presented visually via Nomogram plots. Model discrimination was evaluated by Harrell’s C index, tROC, and tAUC. The calibration of the model was assessed by calibration curves. In addition, the clinical utility of the model was assessed using a DCA curve. After calculating the total risk score of patients in the training set, patients were stratified by risk using percentile partitioning. The Kaplan–Meier method was used to plot OS and PFS survival curves for different risk groups and response statuses at different milestone time points. Differences in survival time groups were compared using the chi-square test. Statistical analysis software included R 4.1.2 and SPSS 26.

**Results:**

This study included a total of 113 ES-SCLC patients who received immunotherapy, including 79 in the training set and 34 in the validation set. Six variables associated with poorer OS in patients were screened by Cox regression analysis: liver metastasis (*P* = 0.001), bone metastasis (*P* = 0.013), NLR < 2.14 (*P* = 0.005), LIPI assessed as poor (*P* < 0.001), PNI < 51.03 (*P* = 0.002), and LDH ≥ 146.5 (*P* = 0.037). A prognostic model for immunotherapy in ES-SCLC patients was constructed based on the above variables. The Harrell’s C-index in the training and validation sets of the model was 0.85 (95% CI 0.76–0.93) and 0.88 (95% CI 0.76–0.99), respectively; the AUC values corresponding to 12, 18, and 24 months in the tROC curves of the training set were 0.745, 0.848, and 0.819 in the training set and 0.858, 0.904 and 0.828 in the validation set; the tAUC curves show that the overall tAUC is > 0.7 and does not fluctuate much over time in both the training and validation sets. The calibration plot demonstrated the good calibration of the model, and the DCA curve indicated that the model had practical clinical applications. Patients in the training set were categorized into low, intermediate, and high risk groups based on their predicted risk scores in the Nomogram graphs. In the training set, 52 patients (66%) died with a median OS of 15.0 months and a median PFS of 7.8 months. Compared with the high-risk group (median OS: 12.3 months), the median OS was significantly longer in the intermediate-risk group (median OS: 24.5 months, HR = 0.47, *P* = 0.038) and the low-risk group (median OS not reached, HR = 0.14, *P* = 0.007). And, the median PFS was also significantly prolonged in the intermediate-risk group (median PFS: 12.7 months, HR = 0.45,* P* = 0.026) and low-risk group (median PFS not reached, HR = 0.12, *P* = 0.004) compared with the high-risk group (median PFS: 6.2 months). Similar results were obtained in the validation set. In addition, we observed that in real-world ES-SCLC patients, at 6 weeks after immunotherapy, the median OS was significantly longer in responders than in non-responders (median OS: 19.5 months vs. 11.9 months, *P* = 0.033). Similar results were obtained at 12 weeks (median OS: 20.7 months vs 11.9 months, *P* = 0.044) and 20 weeks (median OS: 20.7 months vs 11.7 months, *P* = 0.015). Finally, we found that in the real world, ES-SCLC patients without liver metastasis (*P* = 0.002), bone metastasis (*P* = 0.001) and a total number of metastatic organs < 2 (*P* = 0.002) are more likely to become long-term survivors after receiving immunotherapy.

**Conclusion:**

This study constructed a new prognostic model based on basic patient clinical characteristics and peripheral blood indices, which can be a good predictor of the prognosis of immunotherapy in ES-SCLC patients; in the real world, the response status at milestone time points (6, 12, and 20 weeks) can be a good indicator of long-term survival in ES-SCLC patients receiving immunotherapy.

## Introduction

Recently, significant success has been observed in the treatment of small cell lung cancer with both international Programmed cell death ligand-1 (PD-L1) inhibitors, such as atezolizumab and durvalumab, and localized Programmed cell death protein-1 (PD-1)/PD-L1 inhibitors, such as serplulimab and adebrelimab (Horn et al. 2018; Paz-Ares et al. 2019; Cheng et al. 2022; Wang et al. 2022) However, not all patients benefit from immunotherapy. Therefore, identifying suitable immune indicators for precise screening of immunotherapy recipients has become a major concern for medical professionals.

Exploring biomarkers for immunotherapy is challenging due to the complex immune traits of small cell lung cancer (SCLC). Research suggests that certain conventional biomarkers like PD-L1 expression and Tumor mutational burden (TMB) are not significantly associated with the success of immuno-combination chemotherapy in SCLC and have yet to be proven effective predictors (Paz-Ares et al. 2019; Reck et al. 2019; Antonia et al. 2016). As research on SCLC variants has progressed, molecular variants of SCLC-Y/SCLC-I have emerged as promising indicators for SCLC immunotherapy (Gay et al. 2021). However, these biomarkers typically require invasive tests dependent on sufficient tumor tissue samples and sophisticated genome sequencing or histopathology methods. Additionally, their high cost limits their clinical use. Moreover, individual markers have limited predictive power which necessitates an increased clinical demand for affordable non-intrusive biomarkers readily available to collectively determine the effectiveness of immunotherapy in SCLC treatment. Consequently, developing a comprehensive predictive model has become a key research avenue towards achieving precise immunotherapy.

The incidence of SCLC is strongly correlated with smoking behavior, which tends to be higher in the northernmost populations of China. Moreover, recent research (Johal et al. 2022; Xie et al. 2023) has identified several predictive factors in SCLC, including the Eastern Cooperative Oncology Group Performance Status (ECOG) score, LDH level, and treatment efficacy. Additionally, the overall inflammatory response plays a crucial role in determining clinical outcomes for SCLC patients (Liu et al. 2023a). Reports (Liu et al. 2023a; Yang et al. 2023; Zhao et al. 2023) suggest that certain blood-related inflammatory markers such as Neutrophil-to-lymphocyte ratio (NLR), Prognostic nutritional index (PNI), and Lung immune prognostic index (LIPI) may impact the survival of SCLC patients. This retrospective study examined survival data, pre-treatment inflammation markers, and various clinicopathological features of 113 extensive-stage small cell lung cancer (ES-SCLC) patients at Harbin Medical University Cancer Hospital. Initially, we developed a comprehensive predictive model to estimate survival rates for ES-SCLC patients undergoing immunotherapy. Furthermore, we confirmed the association between treatment response at milestone time points (e.g., 6 weeks, 12 weeks, and 20 weeks) and prolonged survival in ES-SCLC patients receiving immunotherapy. Finally, we analyzed differences in clinical characteristics between long-term survivors (LTS) undergoing immunotherapy for ES-SCLC and those who did not survive long term. This research aims to provide insights for personalized treatments tailored to ES-SCLC patients and enhance clinical approaches to immunotherapy.

## Materials and methods

### Object of study

**Fig. 1:**
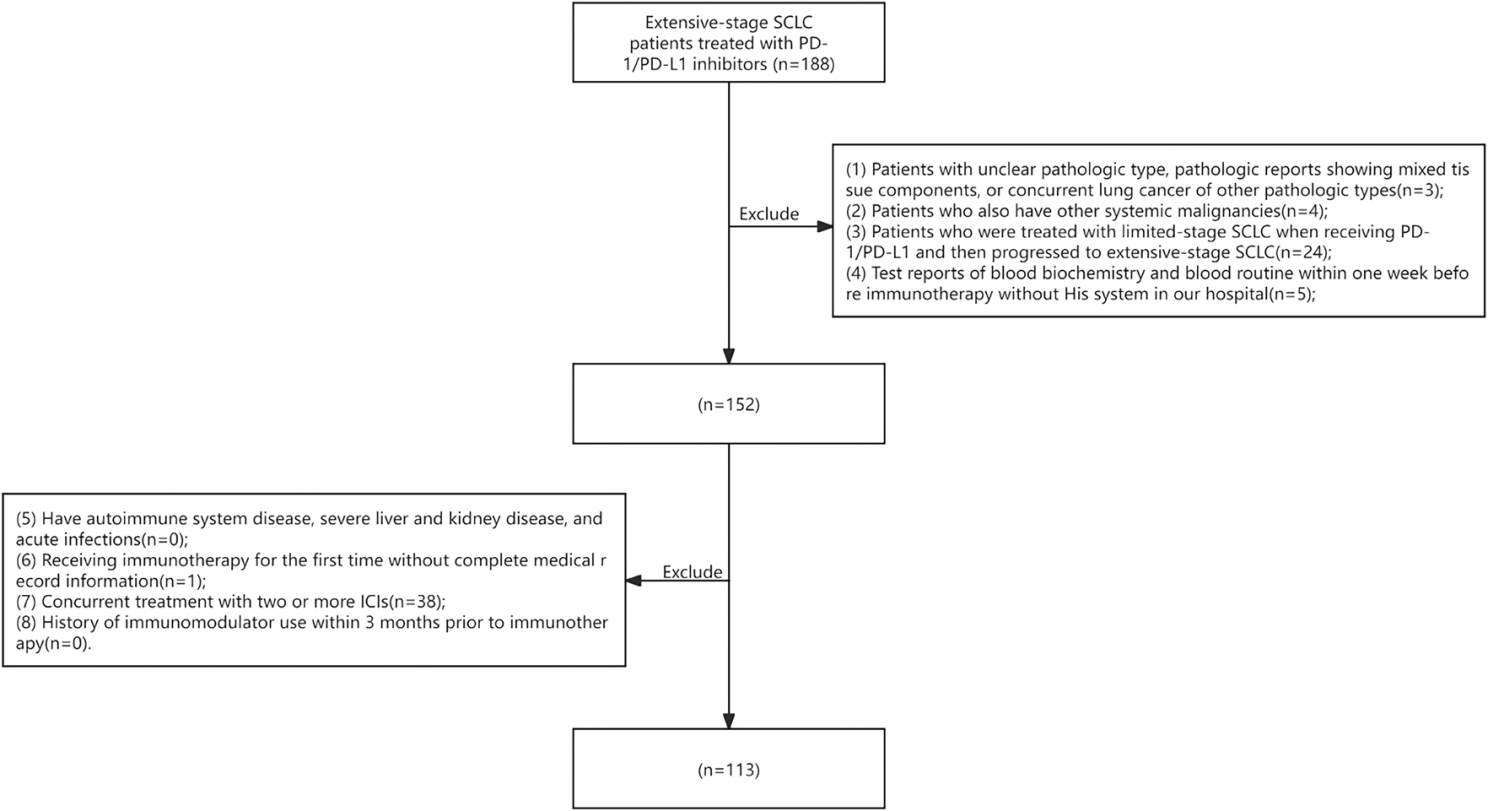
Flow chart

The data for this study were collected from 113 individuals with ES-SCLC who underwent initial treatment with PD-1/PD-L1 inhibitors at the Harbin Medical University Cancer Hospital between March 1, 2019, and October 31, 2022. The follow-up period extended until October 31, 2023. Inclusion and exclusion criteria are provided below, along with the screening flowchart (Fig. 1).

### Inclusion criteria

1. Patients diagnosed with extensive stage small cell lung cancer based on histopathology and imaging at our institution, following the 8th edition of the TNM staging criteria for lung cancer developed by the American Joint Committee on Cancer in conjunction with the American Legion Lung Cancer Association’s Stage II staging method.
2. Patients who received PD-1/PD-L1 inhibitor treatment at our institution for a minimum of two cycles.
3. Hematology tests conducted within one week prior to immunotherapy at our institution.
4. Must have at least one measurable lesion identified as the target lesion.
5. Complete medical records available from initial immunotherapy at our hospital.

### Exclusion criteria

1. Patients with an indeterminate histological subtype, pathological reports indicating mixed tissue components, or concurrent lung cancer of different histological subtypes.
2. Other systemic malignancies.
3. Patients who were initially treated for limited-stage SCLC and subsequently progressed to extensive-stage SCLC while receiving PD-1/PD-L1 therapy.
4. Test reports of blood biochemistry and blood routine within one week prior to immunotherapy without the availability of the His system in our hospital.
5. Autoimmune diseases, acute infections, or severe liver and kidney disorders.
6. Insufficient medical records available for patients’ initial receipt of immunotherapy.
7. Receiving two or more immune checkpoint inhibitors (ICIs) simultaneously.
8. History of using immunomodulators within three months prior to initiating immunotherapy.

### Gathering and defining clinical data

#### Basic clinical characteristics

The basic clinical characteristics were collected at the initiation of ICIs treatment, encompassing age, gender, ECOG performance status, height, weight, smoking history, family history of malignancy, presence of liver metastases, bone metastases and brain metastases status, number of metastatic organs involved, presence of superior vena cava syndrome and duration from first ICI use. Based on the aforementioned data set:

1. Body mass index (BMI) = weight/height.^2^ (weight in kilograms, height in meters)
2. Smoking index = number of cigarettes smoked per day × number of years smoked.

#### Laboratory-related indicators

The patient’s laboratory-related metrics, including white blood cell counts, platelet counts, lymphocytes, and neutrophils in routine blood cell tests, as well as low levels of LDH, serum albumin, sodium, and chloride ions in blood biochemistry were collected one week prior to the initiation of ICIs treatment. Based on the aforementioned data, relevant composite indices were calculated as follows:

1. Neutrophil–lymphocyte ratio (NLR) = Neutrophil count (10^9^/L)/lymphocyte count (10^9^/L)
2. Derived neutrophil–lymphocyte ratio (dNLR) = Neutrophil count (10^9^/L)/(leukocyte-neutrophil count) (10^9^/L)
3. Platelet to lymphocyte ratio (PLR) = platelet count (10^9^/L)/lymphocyte count (10^9^/L)
4. Prognostic Nutritional Index (PNI) = 10 × albumin (g/L) + 0.05 × lymphocyte count (10^9^/L)
5. The Lung Immune Prognostic Index (LIPI) evaluation is based on the following prognostic measures: a dNLR equal to or exceeding 3 and an LDH level equal to or surpassing the upper limit of normal. Subsequently, patients were categorized into three groups based on these factors: good (0 factors), mediam (1 factor), and poor (2 factors).

#### Endpoints and assessment

The efficacy of patients was assessed using the Solid Tumor Efficacy Criteria version 1.1. The primary objective of this study was to determine Overall Survival (OS), which is defined as the duration from the initial administration of ICIs until death from any cause or until reaching the follow-up limit. Additionally, progression-free survival (PFS), defined as the period from the first ICI treatment to disease progression or patient death due to any cause or follow-up limit, served as a secondary endpoint. Patient response statuses were recorded at different time points according to the following definitions:

1. *Complete response (CR)*: Disappearance of all tumor target lesions, absence of new lesions, and normalization of tumor markers for at least 4 weeks.
2. *Partial response (PR)*: ≥ 30% reduction in sum of largest diameters of tumor target lesions for at least 4 weeks.
3. *Stable disease (SD)*: Decrease in sum of largest diameters of target lesions without meeting PR criteria or increase without meeting progressive disease (PD) criteria.
4. *Progressive disease (PD)*: ≥ 20% increase in sum of largest diameters of tumor target lesions or appearance of new lesions.

### Model construction and assessment methods

#### Model construction

The study involved the random allocation of 113 patients with ES-SCLC in a ratio of 7:3, where 79 patients (accounting for 70% of the total) were used to construct the model (training set), and 34 patients (30% of the total) were used to validate the model (validation set). Factors associated with immunotherapy prognosis were analyzed using Univariate analysis (*P* < 0.05). Furthermore, these significant factors were incorporated into a comprehensive Cox regression analysis along with relevant studies to refine variable selection for modeling purposes. Subsequently, a predictive model was developed to estimate survival probabilities for patients undergoing immunotherapy for ES-SCLC, and column line graphs were generated to illustrate survival chances at 12, 18, and 24 months.

#### Model assessment methods

The model’s differentiation assessment employed Harrell’s C-index, Time-dependent Receiver Operating Characteristic Curve (tROC) and Time-dependent Area Under Curve (tAUC). The calibration of the model was assessed using a Calibration curve. Additionally, clinical utility of the model was evaluated through Clinical Decision Curve Analysis (DCA). Patients were stratified into risk groups based on percentile segmentation.

### Statistical methods

The Cox regression method was employed for both univariate and multivariate analyses. Various interpolation methods were utilized to fill the gaps in the data. Survival trajectories for OS and PFS were plotted at distinct intervals across different risk tiers and response stages using the Kaplan–Meier method. Differences in survival duration among patient categories were evaluated using the Chi-square test. Statistical analyses were conducted using R 4.1.2 and SPSS 26, with a significance level set at p < 0.05.

## Results

### Baseline patient characteristics

**Fig. 2:**
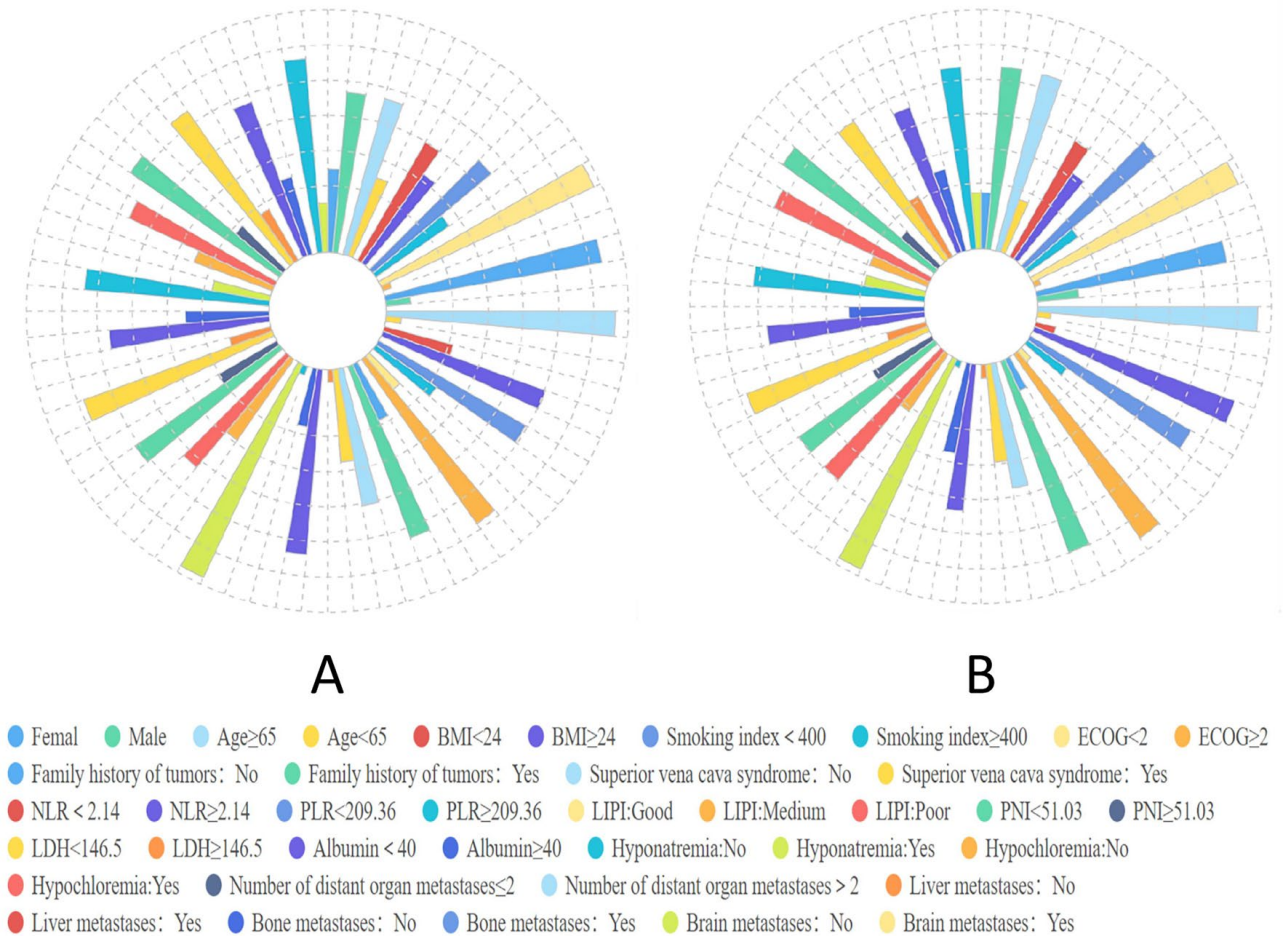
Visualized presentation of the overall categorical clinical features from the (A) train dataset and (B) test dataset

The study recruited 113 participants diagnosed with ES-SCLC, and Table 1 presents the baseline characteristics of the training, validation, and patient cohorts. The clinical features are depicted in Fig. 2. Among the patients, the mean age was 61 years; 78 (69%) were male; 38 (34%) had a smoking index of ≥ 400; 14 (12%) had a family history of tumors; all patients with an ECOG score < 2 in 109 (96%) cases; superior vena cava syndrome was observed in 7 (6%); liver metastases were present in 29 (26%); bone metastases were detected in 38 (34%); brain metastases occurred in 24 (21%); and majority, i.e.,86(76%), had fewer than two affected organ sites. The ICIs utilized for this investigation included atezolizumab, durvalumab, and serplulimab. By the end of the follow-up period, mortality was recorded among 74 (65%) patients while 39 (35%) survived.

**Table 1:** Baseline characteristics of patients

Characteristic	Train set, N = 79 (%)	Validation set, N = 34 (%)	Pooled cohort, N = 113 (%)	*P*
Gender				0.273
Female	24 (30%)	9 (26%)	35 (31%)	
Male	55 (70%)	25 (74%)	78 (69%)	
Age				0.814
≥ 65	50 (63%)	28 (82%)	78 (69%)	
< 65	29 (37%)	6 (18%)	35 (31%)	
BMI				0.787
< 24	41 (52%)	21 (62%)	62 (55%)	
≥ 24	38 (48%)	13 (38%)	51 (45%)	
Smoking index				0.851
< 400	52 (66%)	23 (68%)	75 (66%)	
≥ 400	27 (34%)	11 (32%)	38 (34%)	
ECOG				0.582
< 2	77 (97%)	32 (94%)	109 (96%)	
≥ 2	2 (3%)	2 (6%)	4 (4%)	
Family history of tumors				0.756
No	70 (89%)	29 (85%)	99 (88%)	
Yes	9 (11%)	5 (15%)	14 (12%)	
Superior vena cava syndrome				1.000
No	74 (94%)	32 (94%)	106 (94%)	
Yes	5 (6%)	2 (6%)	7 (6%)	
NLR				0.931
< 2.14	20 (25%)	6 (18%)	26 (23%)	
≥ 2.14	59 (75%)	28 (82%)	87 (77%)	
PLR				0.880
< 209.36	52 (66%)	30 (88%)	82 (73%)	
≥ 209.36	27 (34%)	4 (12%)	31 (27%)	
LIPI				0.716
Good	43 (54%)	20 (59%)	63 (56%)	
Medium	34 (43%)	10 (29%)	44 (39%)	
Poor	2 (3%)	4 (12%)	6 (5%)	
PNI				0.125
< 51.03	57 (72%)	24 (71%)	81 (72%)	
≥ 51.03	22 (28%)	10 (29%)	32 (28%)	
LDH				1.000
< 146.5	3 (4%)	1 (3%)	4 (4%)	
≥ 146.5	76 (96%)	33 (97%)	109 (96%)	
Albumin				0.153
< 40	30 (38%)	12 (35%)	42 (37%)	
≥ 40	49 (62%)	22 (65%)	71 (63%)	
Hyponatremia				0.532
No	55 (70%)	26 (76%)	81 (72%)	
Yes	24 (30%)	8 (24%)	32 (28%)	
Hypochloremia				0.287
No	63 (80%)	30 (88%)	93 (82%)	
Yes	16 (20%)	4 (12%)	20 (18%)	
Number of distant organ metastases				0.589
< 2	59 (75%)	27 (79%)	86 (76%)	
≥ 2	20 (25%)	7 (21%)	27 (24%)	
Liver metastases				0.733
No	61 (77%)	23 (68%)	84 (74%)	
Yes	18 (23%)	11 (32%)	29 (26%)	
Bone metastases				0.806
No	53 (67%)	22 (65%)	75 (66%)	
Yes	26 (33%)	12 (35%)	38 (34%)	
Brain metastases				0.106
No	64 (81%)	25 (74%)	89 (79%)	
Yes	15 (19%)	9 (26%)	24 (21%)

### Optimal cut-off values for continuity variables in SCLC patients

**Fig. 3:**
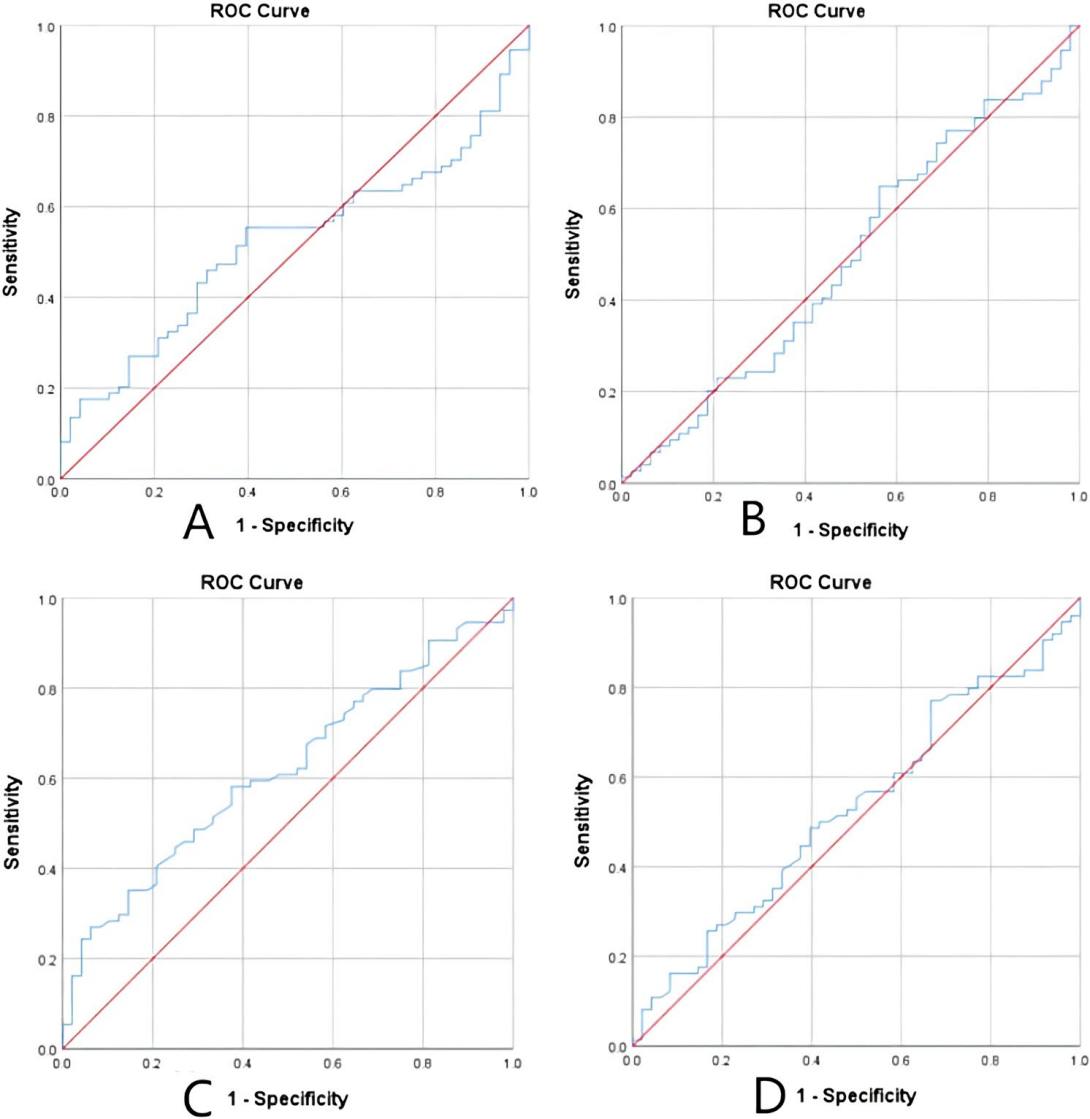
ROC curve of NLR (**A**), PLR (**B**), LDH (**C**) PNI (**D**)

Leveraging relevant studies and clinical significance, we utilized threshold values to discretize continuous variables into binary variables. The selected thresholds for serum albumin, hyponatremia, and hypochloremia were based on the lowest normal reference in our blood biochemistry tests (serum albumin = 40 g/L, hyponatremia NA^+^  < 137 mmol/L, hypochloremia Cl^−^ < 99 mmol/L). Additionally, we set the smoking index at 400, ECOG score at 2, number of metastatic organs at 2, and BMI threshold at 24. To accurately determine the optimal thresholds for NLR, PLR, LDH, and PNI parameters in our study Receiver Operating Characteristic Curve (ROC), Area Under Curve (AUC), and Jordon’s index were employed (Fig. 3). NLR was found to be equal to 2.14 while PLR was calculated as 209.36; LDH had a value of 146.5; PNI was measured as being equal to51.03.

### Filtering variable results

**Fig. 4:**
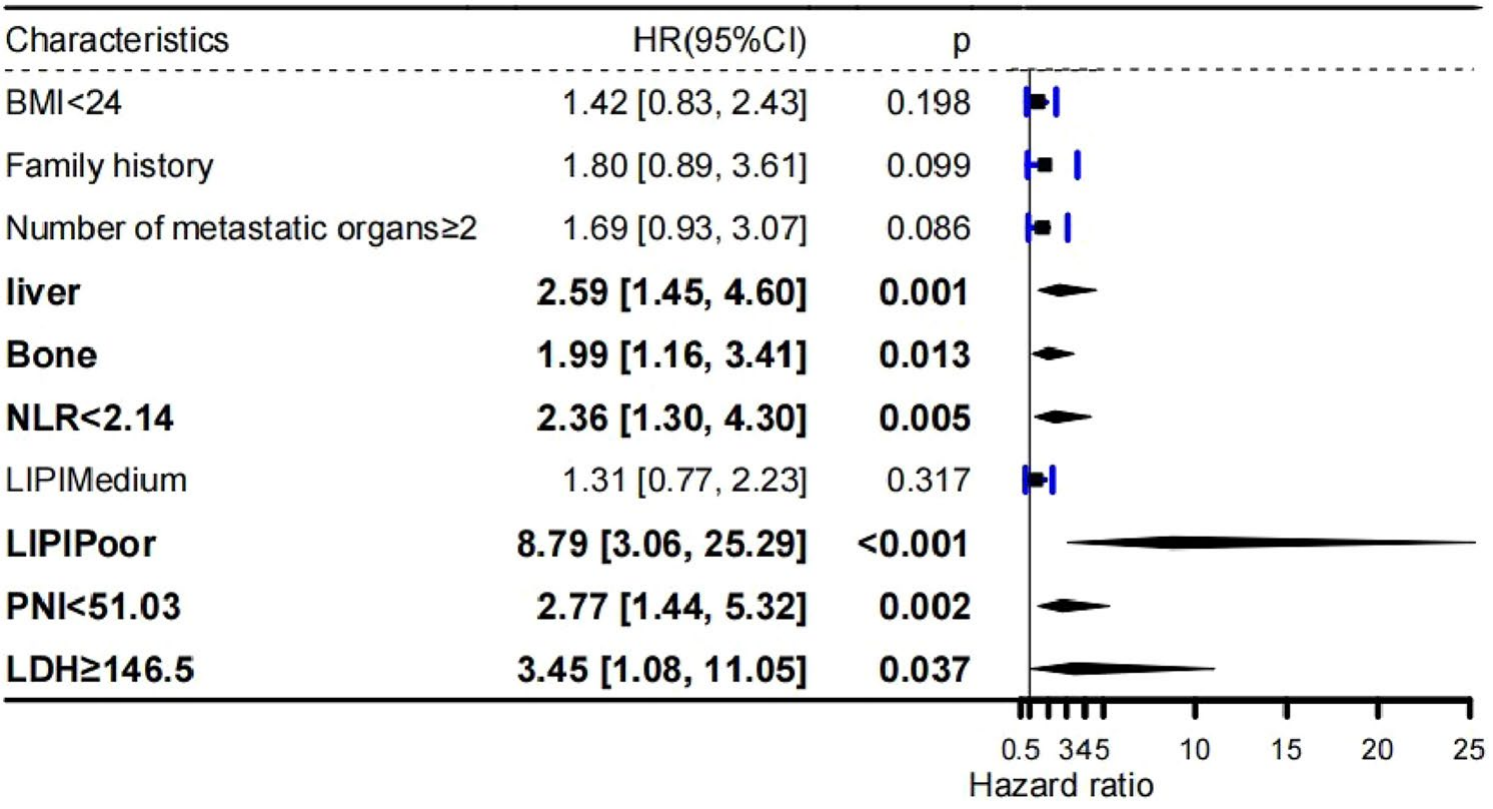
Multivariate Cox regression forest map

In this study, Cox regression analysis was employed for variable screening. Initially, our findings indicated that a blood test NLR < 2.14 and LIPI deemed subpar, along with a BMI < 24, familial tumor history, liver and bone metastases, and the count of distant organ metastases ≥ 2 in clinical features were significantly associated with reduced OS according to univariate Cox regression analysis (*P* < 0.05) (Table 2). Meanwhile, existing literature and clinical experience have demonstrated the prognostic significance of LDH and PNI in patients with ES-SCLC. Subsequent multifactorial Cox regression analysis of these factors led to the exclusion of six indicators ultimately linked to a worse prognosis: liver and bone metastases, NLR < 2.14, poor LIPI rating, PNI < 51.03, and LDH ≥ 146.5 (Fig. 4).

**Table 2:** Univariate analysis

Characteristic	Hazard ratio (95% CI)	*P* value
Gender		
Male	1	
Female	0.697 (0.416–1.165)	0.169
BMI		
< 24	1	
≥ 24	0.592 (0.365–0.959)	0.033
Family history of tumors		
No	1	
Yes	2.171 (1.157–4.073)	0.016
Number of distant organ metastases		
< 2	1	
≥ 2	2.206 (1.332–3.652)	0.002
Liver metastases		
No	1	
Yes	2.808 (1.729–4.56)	0
Bone metastases		
No	1	
Yes	2.664 (1.661–4.273)	0
Brain metastases		
No	1	
Yes	0.717 (0.385–1.333)	0.293
NLR		
< 2.14	1	
≥ 2.14	0.505 (0.304–0.837)	0.008
PLR		
< 209.36	1	
≥ 209.36	0.795 (0.467–1.356)	0.401
LIPI		
Good	1	
Medium	1.601 (0.994–2.582)	0.054
Poor	3.932 (1.531–10.141)	0.005
PNI		
< 51.03	1	
≥ 51.03	1.553 (0.901–2.679)	0.113
LDH		
< 146.5	1	
≥ 146.5	0.396 (0.142–1.102)	0.076

### Model construction

**Fig. 5:**
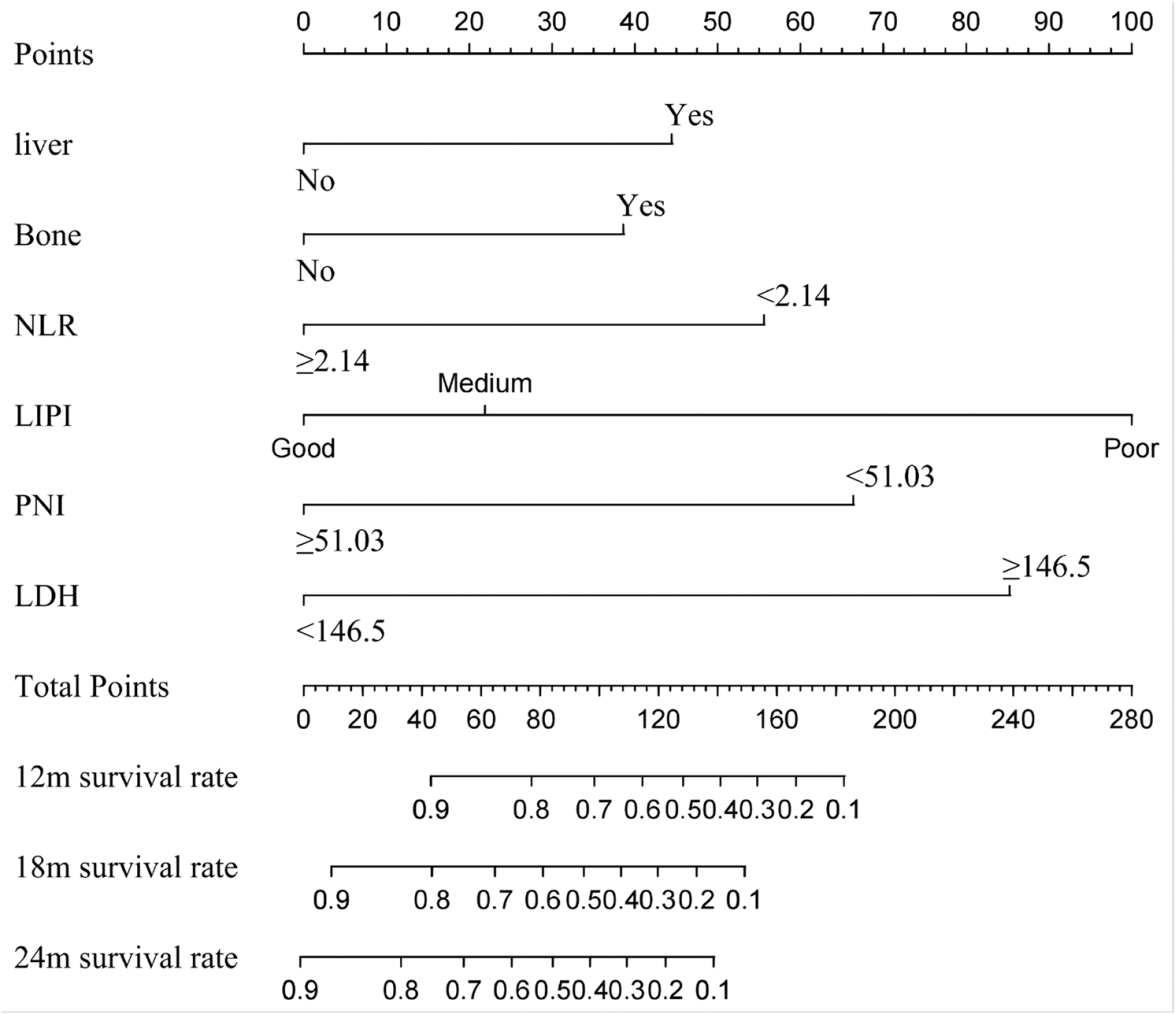
Multivariate analysis of train set

The probability of predicting patient survival at 12, 18, and 24 months was visualized using a Nomogram plot based on the six variables included in the final model (Fig. 5). The respective scores for each variable on the Nomogram plot were as follows: liver metastasis—score: 45, bone metastasis—score: 38, NLR < 2.14—score: 56, LIPI assessment (good—score: 0; medium—score: 22; poor—score: 99), PNI < 51.03–55, and LDH ≥ 146.5–87 (refer to Table 3). The overall patient score represents an aggregate of their individual scores for each variable, with corresponding forecasted survival probabilities available in the column line graph for the periods of interest (i.e.,12-months,18-months, and24-months) based on these scores.

**Table 3:** The risk scoring system

Variable	Liver		Bone		NLR		LIPI			PNI		LDH	
	No	Yes	No	Yes	≥ 2.14	< 2.14	Good	Medium	Poor	≥ 51.03	< 51.03	≥ 146.5	< 146.5
Score	0	45	0	38	0	56	0	22	99	0	66	87	0

Risk stratification Low risk: 0–69; Medium risk: 70–162; High risk: ≥ 163

### Evaluation of model effectiveness and validation

**Fig. 6:**
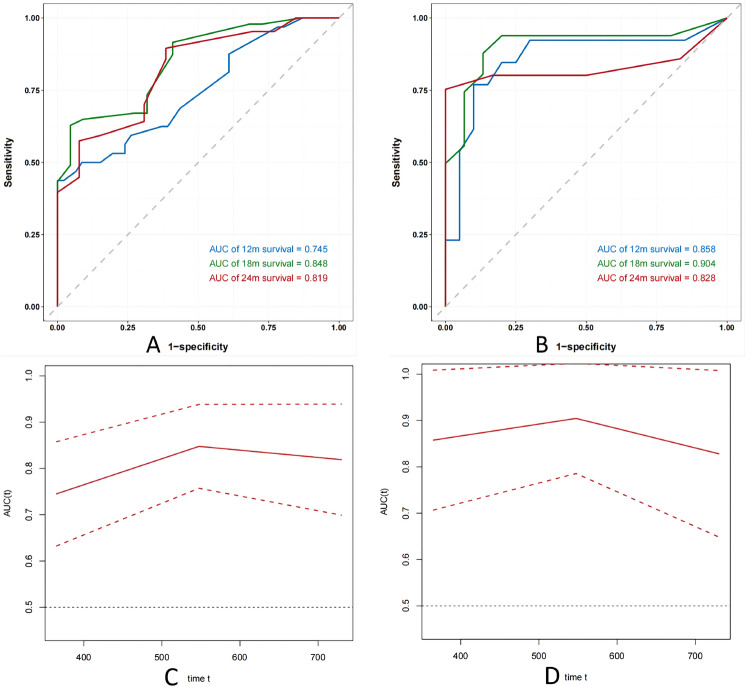
**A** 12/18/24-month ROC curve of train set, **B** 12/18/24-month ROC curve of validation set, **C** tAUC of train set, **D** tAUC of validation set

The accuracy of the model predictions was assessed using differentiation and calibration methods in this study. Harrell’s C-index, tROC curve, and tAUC curve were employed to evaluate the discriminative ability of the model. For both the training and validation datasets, Harrell’s C-index scores were 0.85 (95% CI: 0.76–0.93) and 0.88 (95% CI 0.76–0.99), respectively, indicating successful model differentiation. Figures (Fig. 6 A and B) demonstrate that at 12, 18, and 24 months, AUC values for the training set were 0.745, 0.848, and 0.819 respectively; while for the validation set they were found to be higher at 0.858, 0.904, and 0.828 respectively. With the exception of the AUC value at month twelve in the training set, all other AUC values consistently exceeded 0.8, indicating significant differentiation by our model. The tAUC depicted in Figures (Fig. 6C and D) illustrates time on the horizontal axis and corresponding AUC values on the vertical axis for both training and validation sets. Overall, tAUC consistently surpassed 0.7, exhibiting slight fluctuations over time while demonstrating effective discrimination capability as well as stability of our prognostic model.

**Fig. 7:**
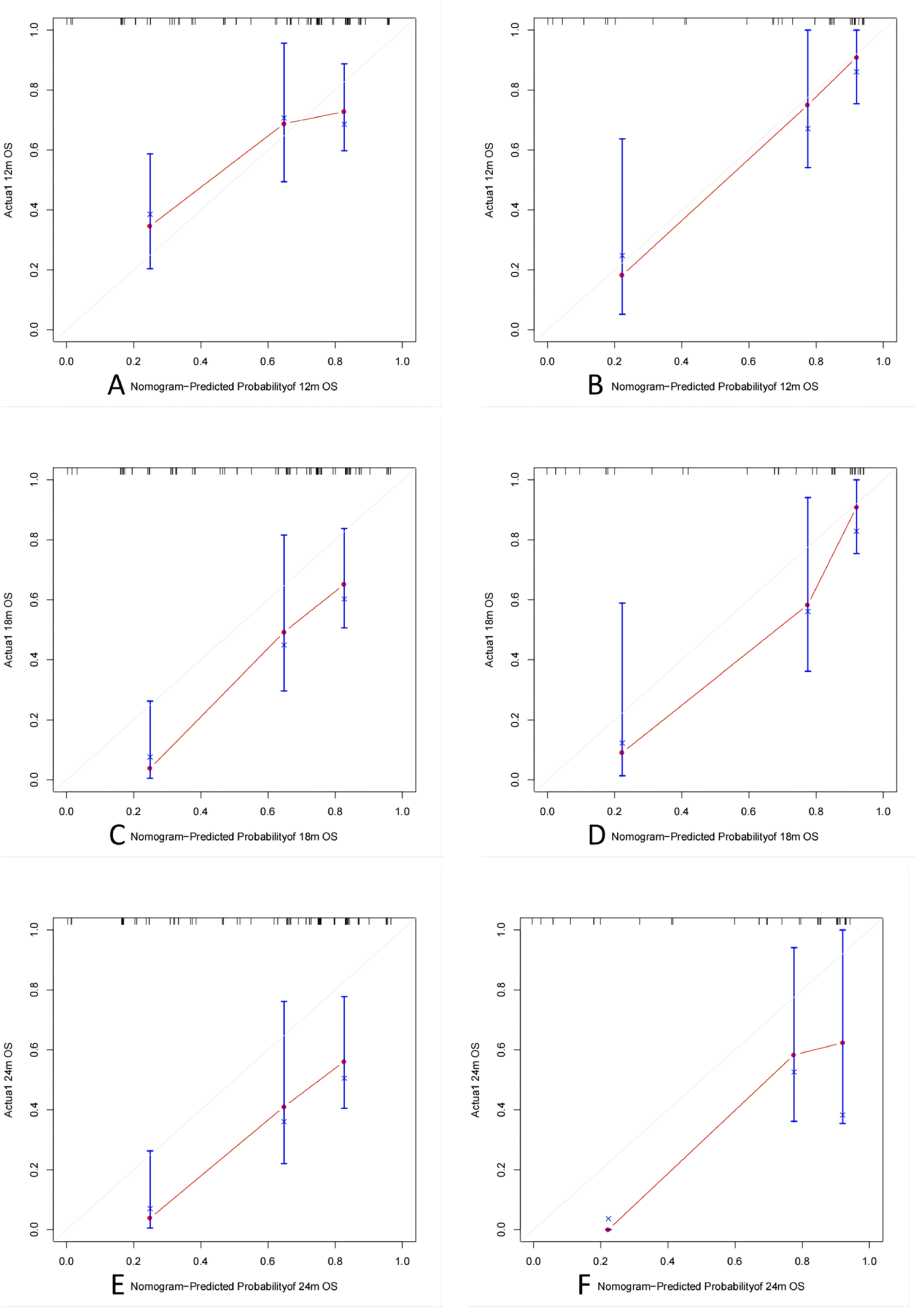
Calibration curves. **A** Train set (12 months); **B** validation set(12 months); **C** train set (18 months); **D** validation set (18 months); **E** train set (24 months); **F** validation set (24 months)

The model’s calibration was assessed by plotting the calibration curve (Fig. 7), which shows the predicted probability of patient death on the x-axis and actual probability of patient death on the y-axis. The diagonal line represents perfect alignment between predicted and actual probabilities. Our model accurately predicts patient mortality at 12 months in both training and validation sets, as well as at 18 months in the validation set. However, for predictions beyond these time points, particularly at 18 and 24 months in the training set and 24 months in the validation set, there is a tendency to overestimate mortality by up to 30% when probability is around 50%. This may be due to several factors: our study has limited long-term data availability due to its relatively short follow-up period; prediction results may exhibit instability because of our small sample size; ES-SCLC patients generally have shorter overall survival times that can impact accuracy when predicting long-term outcomes. It should be noted that our model demonstrates excellent calibration performance.

### Assessment of actual needs for clinical decision-making models

**Fig. 8:**
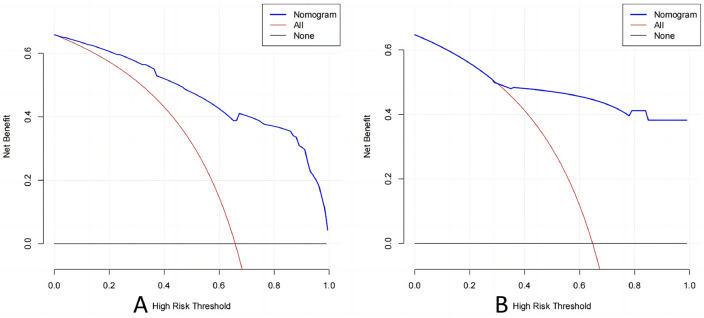
DCA. **A** Train set; **B** validation set

In this study, DCA curves were generated by plotting the 12-month survival probabilities of patients to assess the practical clinical relevance of the model. As depicted in Fig. 8, the x-axis represents the threshold probability, while the y-axis indicates the net benefit rate of the model. The gray line signifies complete mortality at 12 months (i.e., a net benefit rate of 0), whereas the red line denotes full patient survival at 12 months, and the blue line represents the net benefit associated with this particular model. Within a threshold probability range of 1.0, both training and validation sets exhibit blue lines that are significantly distant from these two extreme curves, thereby indicating substantial clinical utility for our current model.

### Survival analysis of patients with different risk stratification

**Fig. 9:**
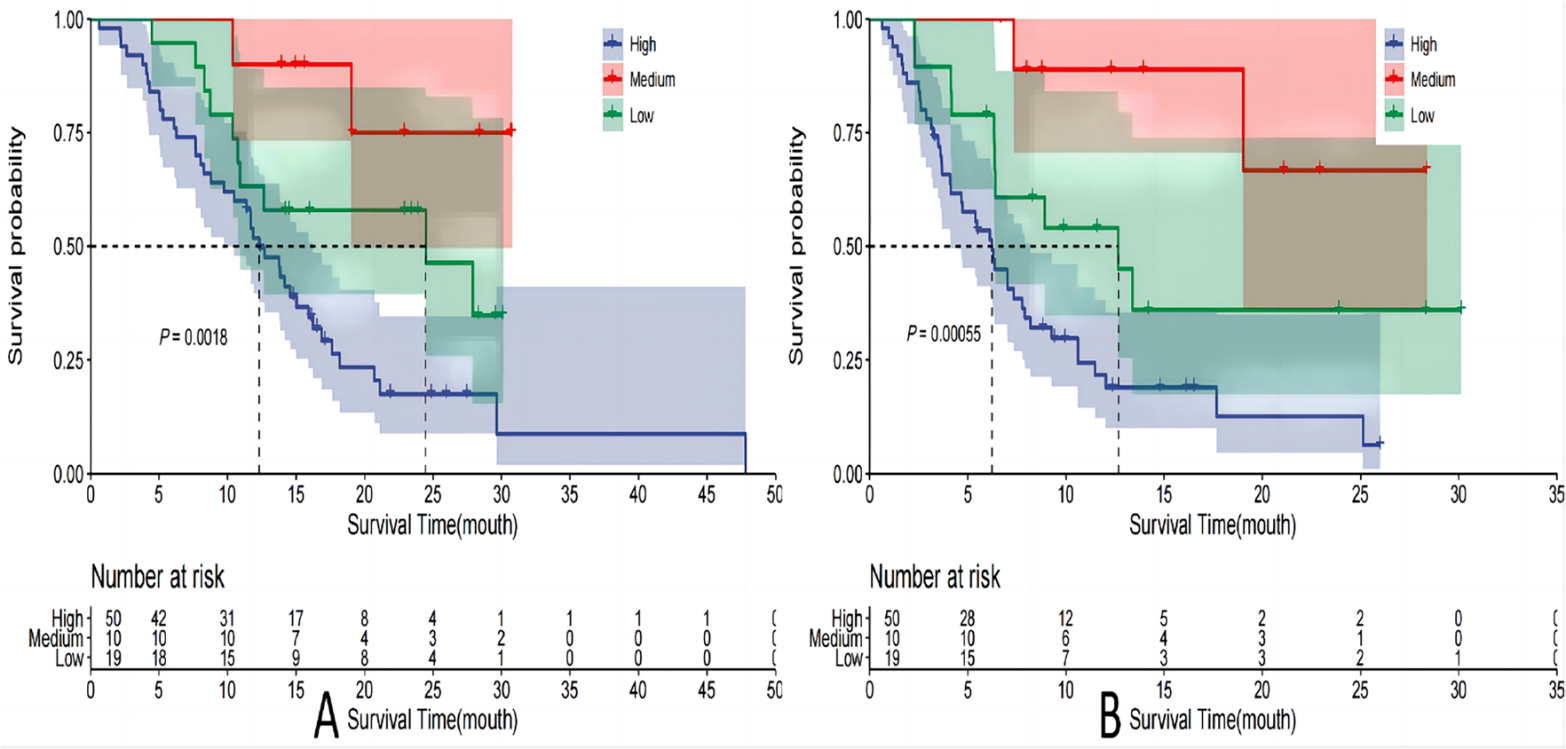
Survival curves of train set. **A** OS; **B** PFS

**Fig. 10:**
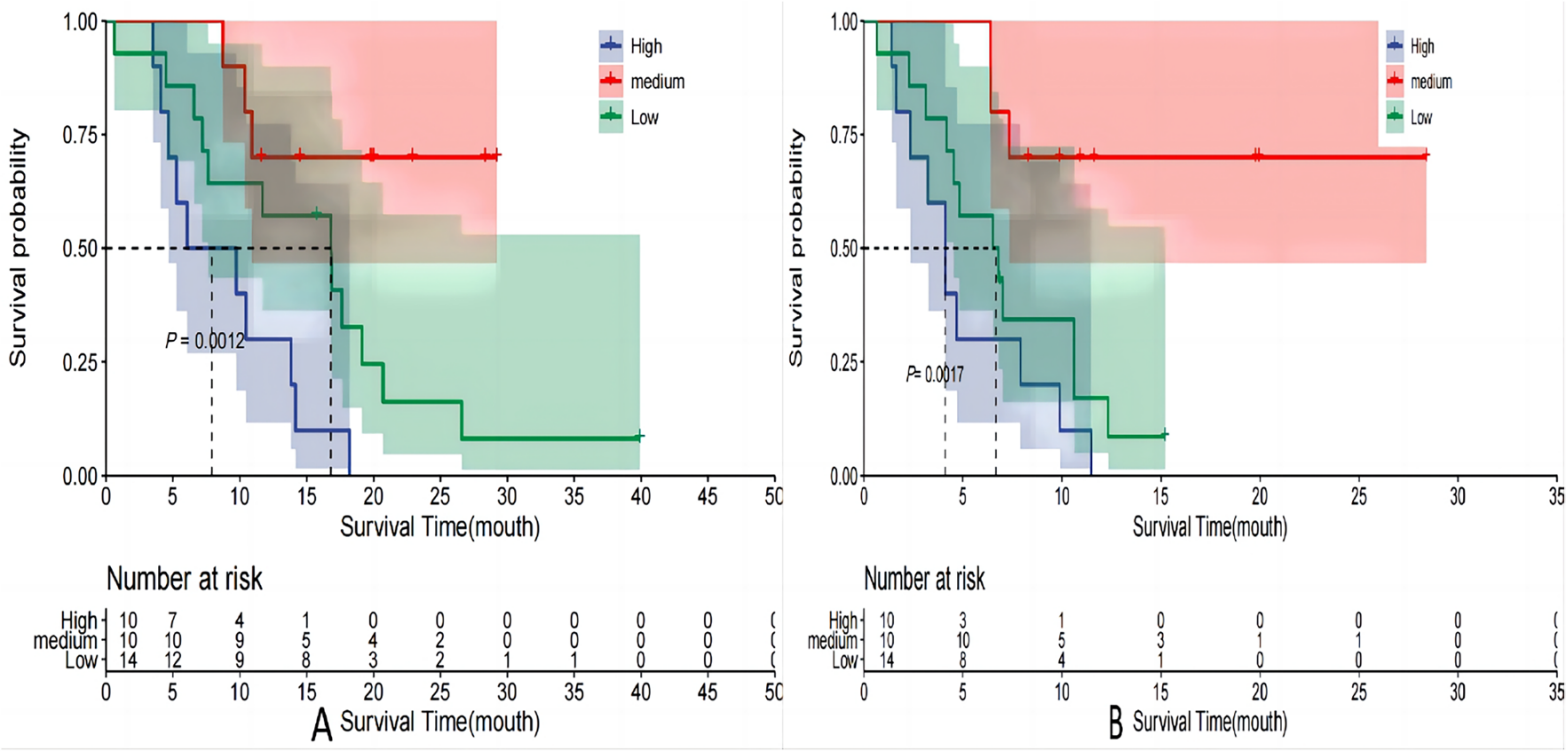
Survival curves of test set. **A** OS; **B** PFS

Patients in the training cohort were stratified based on their predicted risk scores using the 25th and 75th percentile brackets, and subsequently categorized into low-risk (0–69), medium-risk (70–162), and high-risk (≥ 163) groups according to the Nomogram plot. Among the 79 participants in the training cohort, 52 (66%) experienced mortality while disease progression was observed in 67 (85%) individuals based on monitoring data. The median overall survival (mOS) was determined as 15.0 months (95% CI 11.9–20.7), with a median progression-free survival (mPFS) estimated at 7.8 months (95% CI 6.4–12.0). A significant increase in overall survival was observed in both the medium-risk group (mOS, 24.5 months; HR = 0.47, 95% CI 0.23–0.96, *P* = 0.038) and low-risk group (mOS not reached; HR = 0.14, 95% CI 0.03–0.58, *P* = 0.007), compared to the high-risk group (mOS, 12.3 months). Additionally, there was a significant increase in mPFS observed in both the medium-risk group (mPFS: 12.7 months; HR = 0.45, 95% CI 0.23–0.91, *P* = 0.026) and low-risk group (mPFS not reached; HR = 0.12, 95% CI 0.03–0.50, *P* = 0.004), compared to the high-risk group (mPFS: 6.2 months) (Table 4, Fig. 9). Among the validation set consisting of 34 participants, a total of 25 individuals (74%) succumbed to the disease and by the date of follow-up, disease progression was observed in 31 participants (91%). The mOS was 13.8 months (95% CI 10.4–20.7), while the mPFS was 6.9 months (95% CI 4.8–11.5). Notably, an extension in OS was observed within both medium-risk group (mOS, 16.8 months; HR = 0.47, 95% CI 0.17–0.99, *P* = 0.047) and low-risk group (mOS not reached; HR = 0.40, 95% CI 0.03–0.42, *P* = 0.001), compared to the high-risk group (mOS, 7.9 months). Furthermore, when compared to the high-risk group (mPFS, 4.1 months), no significant improvement in mPFS was noted within medium-risk group (mPFS, 6.65 months; HR = 0.56, 95% CI 0.24–1.33, *P* = 0.189), Conversely, the low-risk group demonstrated a significantly prolonged mPFS (mPFS not reached; HR = 0.12, 95% CI 0.03–0.44, *P* = 0.002). (Table 5, Fig. 10).

**Table 4:** OS and PFS based on the risk stratification in train set

Groups	N (%)	OS			PFS		
		mOS(m)	HR (95% CI)	*P*	mPFS(m)	HR (95% CI)	*P*
High	50 (63%)	12.3	1		6.2	1	
Medium	10 (13%)	24.5	0.47 (0.23–0.96)	0.038	12.7	0.45 (0.23–0.91)	0.026
Low	19 (24%)	NA	0.14 (0.03–0.58)	0.007	NA	0.12 (0.03–0.50)	0.004

**Table 5:** OS and PFS based on the risk stratification in test set

Groups	N (%)	OS			PFS		
		mOS (m)	HR (95% CI)	*P*	mPFS (m)	HR (95% CI)	*P*
High	10 (29%)	7.9	1		4.1	1	
Medium	10 (29%)	16.8	0.47 (0.17–0.99)	0.047	6.65	0.56 (0.24–1.33)	0.189
Low	14 (42%)	NA	0.40 (0.03–0.42)	0.001	NA	0.12 (0.03–0.44)	0.002

### The correlation between milestone response status and survival

**Fig. 11:**
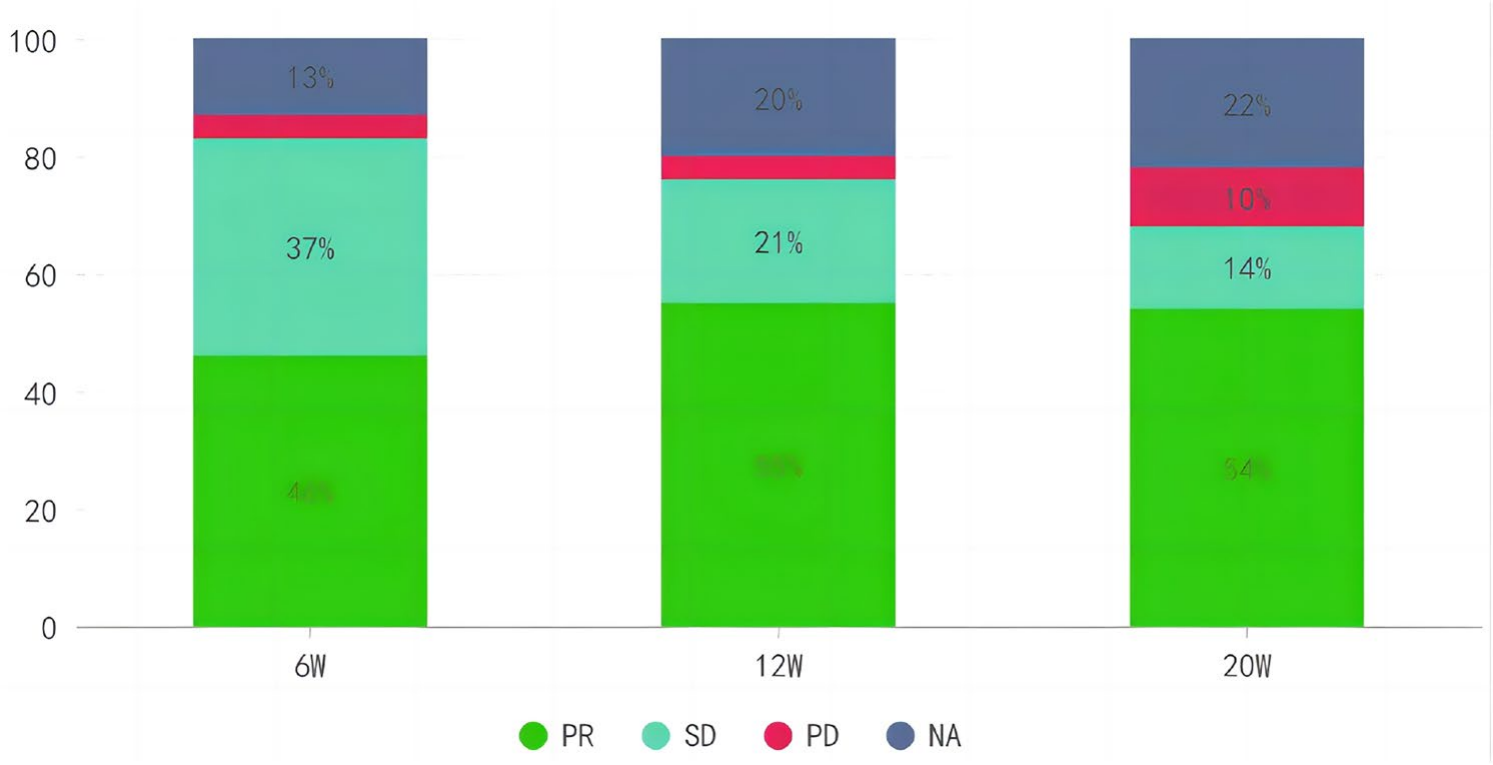
Response status of patients treated with immune checkpoint inhibitors at 6, 12, and 24 weeks

**Fig. 12:**
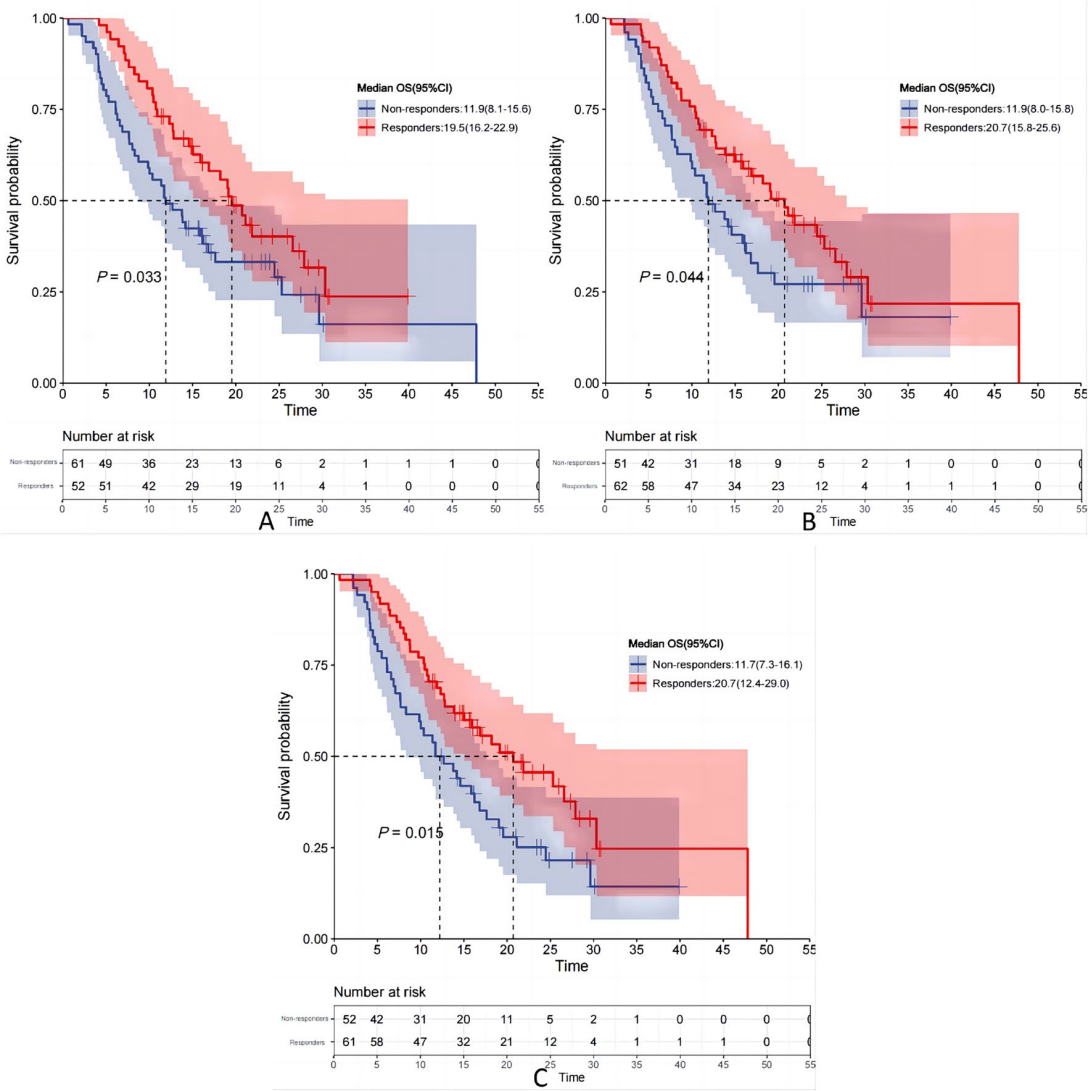
Kaplan–Meier OS curves by response from **A** 6-week. **B** 12-week **C** 20-week landmark

The study further investigated the association between patients’ responses at milestone time points (6, 12, and 20 weeks) and the survival rates of patients treated with immune checkpoint inhibitors during these time points, as illustrated in Fig. 11. In this study, responders were defined as patients who achieved CR or PR at each milestone time point; all other patients were classified as non-responders. At 6 weeks after initiation of immune checkpoint inhibitor treatment, a total of 52 patients exhibited PR, with a response rate of 46%. The relationship between their response and OS is depicted in Fig. 12A. Following 6 weeks of combined therapy with immune checkpoint inhibitors and chemotherapy, responders demonstrated a significant long-term survival advantage compared to non-responders (mOS: 19.5 months vs. 11.9 months; *P* = 0.033). Similar patterns were observed at both the 12 weeks and 20 weeks milestones (refer to Fig. 12B and C), aligning with those seen at 6 weeks, indicating that responders experienced significantly prolonged survival periods compared to non-responders (mOS: 20.7 months vs. 11.9 months; mOS: 20.7 months vs. 11.7 months), supported by statistical significance levels of *P* = 0.044 and *P* = 0.015, respectively.

**Fig. 13:**
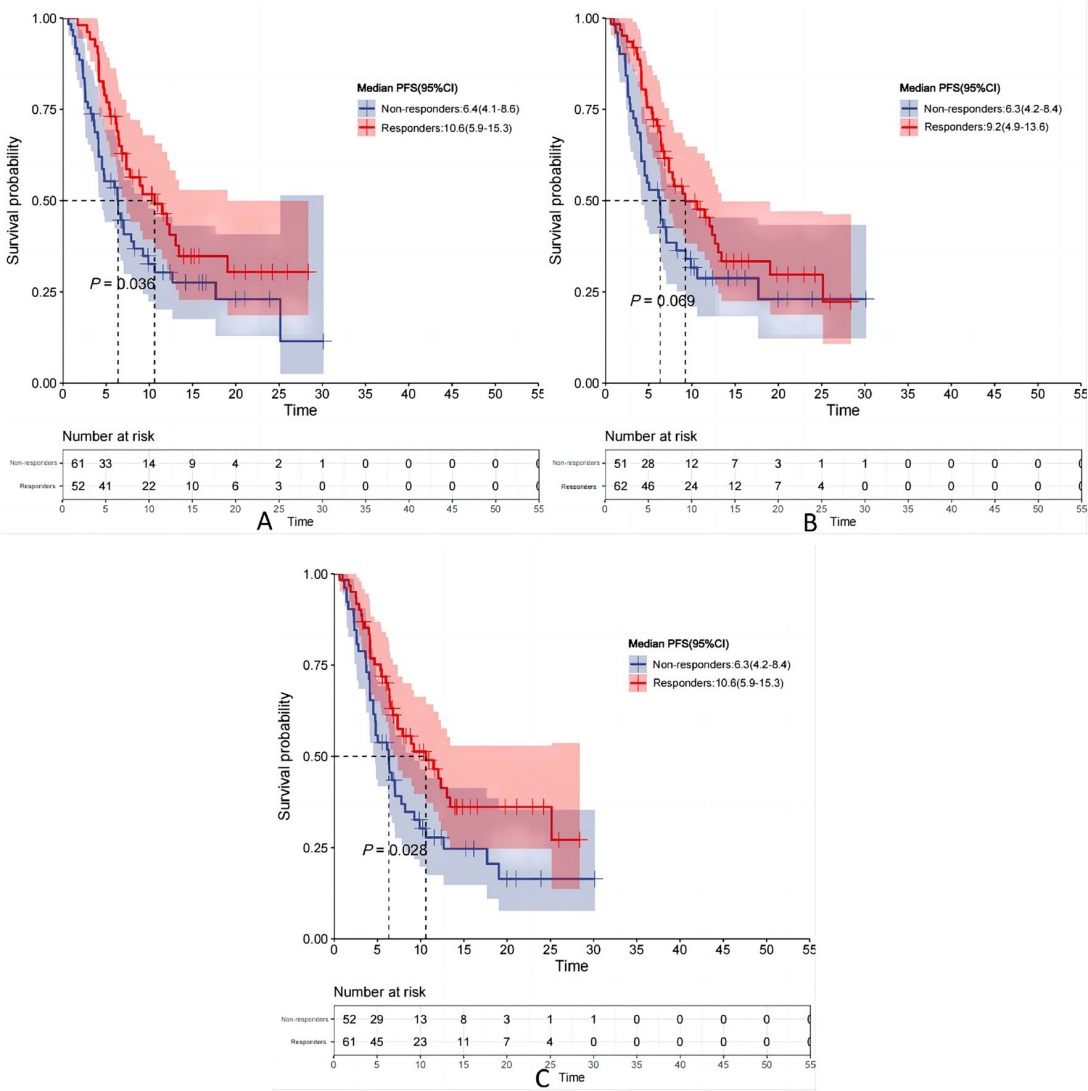
Kaplan–Meier PFS curves by response from **A** 6-week. **B** 12-week **C** 20-week landmark

Additionally, a significant association exists between the patients’ response status and PFS at milestone time points. As depicted in Fig. 13, after 6 and 20 weeks of treatment with immune checkpoint inhibitors combined with chemotherapy, responders exhibited significantly prolonged PFS compared to non-responders, with mPFS of 10.6 months vs. 6.4 months and 10.6 months vs. 6.3 months respectively. These observed differences were statistically significant, as indicated by p-values of 0.036 and 0.028 respectively. Furthermore, at the 12 weeks milestone, responders demonstrated a tendency towards extended survival with a PFS of 9.2 months (95% CI 4.9–13.6), while non-responders had a PFS of only 6.3 months (95% CI 4.2–8.4). However, this disparity did not reach statistical significance (*P* = 0.069).

### Exploratory analysis of the potential clinical characteristics of LTS

Furthermore, our research investigated the potential characteristics of LTS in the treatment of ES-SCLC. The findings demonstrate a 1-year OS rate of 58.4% following immunotherapy in patients with ES-SCLC, closely aligning with the 1-year OS rates observed in numerous extensive clinical trials, as detailed in Table 6. These include a rate of 52% in the A + CE treatment group of the IMpower133 study, 52.8% in the D + EP group of the CASPIAN study, and 62.9% and 60.7% respectively in the immuno-combination chemotherapy groups of CAPSTONE-1 and ASTRUM-005 studies within their respective immunocombination chemotherapy groups. The study’s 18-month survival rate was found to be 33.6%, closely mirroring results from CASPIAN and IMpower133 studies. Moreover, our research revealed a 2-year OS rate of 17.7%, slightly lower than rates observed in other clinical trials; this discrepancy may be attributed to limited participant numbers and a short follow-up period within this study cohort. Nevertheless, these aforementioned data strongly support the reliability and validity of our study.

**Table 6:** OS rate of the various studies

	IMpower133	CASPIAN	CAPSTONE-1	ASTRUM-005	This study
12-month OS rate	52%	52.8%	62.9%	60.7%	58.4%
18-month OS rate	34%	32%	–	–	33.6%
24-month OS rate	22%	22.9%	31.3%	43.1%	17.7%

**Fig. 14:**
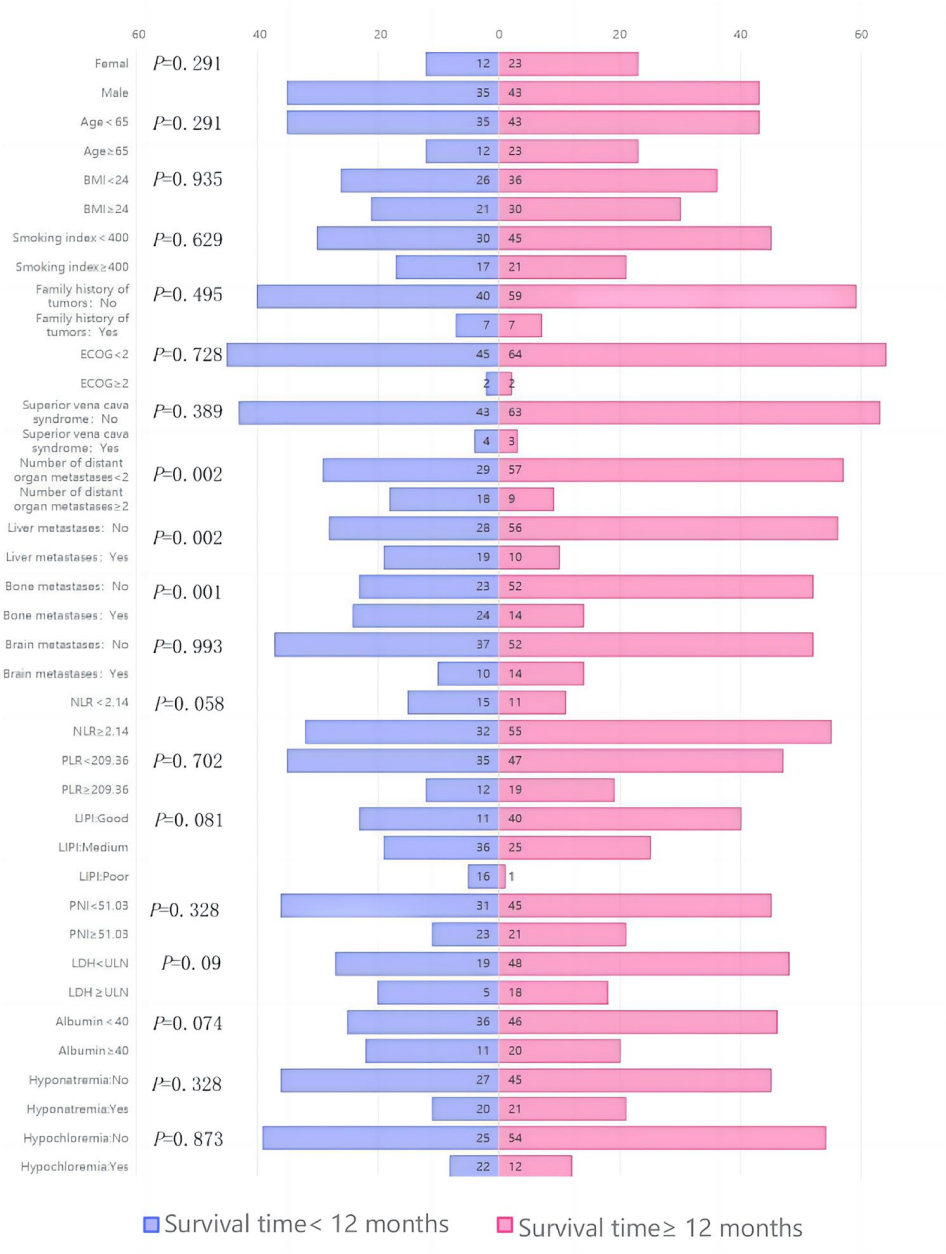
Baseline characteristics of different survival times patients

By investigating the clinical characteristics of patients with prolonged survival times, in conjunction with the previously reported mOS durations (12.9 months in CASPIAN, 12.3 months in IMpower133, 15 months in ASTRUM-005, and 15.3 months in CAPSTONE-1), we categorized patients treated with immunocheckpoint inhibitors based on their survival periods exceeding 12 months. The classification was established using a 12-month threshold, and subsequently subjected to chi-squared test analysis for both patient groups as depicted in Fig. 14. Notably, patients without initial liver or bone metastases and those with fewer than two metastatic organs generally exhibited significantly extended survival times (*P* = 0.002, *P* = 0.001, and *P* = 0.002 respectively).

Meanwhile, our study revealed a tendency towards an enhanced immune response in patients with LDH levels below the upper limit of normal (ULN), NLR values ≥ 2.14, albumin levels ≥ 40 g/L, and higher LIPI scores; however, these differences did not reach statistical significance (*P* = 0.09, 0.058, 0.074, and 0.081).

## Discussion

The utilization of immune checkpoint inhibitors in the treatment of SCLC has yielded numerous clinical trials showcasing the significant efficacy of immune-combination chemotherapy for advanced cases (Horn et al. 2018; Paz-Ares et al. 2019; Cheng et al. 2022; Wang et al. 2022). However, not all patients derive benefits from immunotherapy. In the era of precision therapy, it is crucial to evaluate the impact of immunotherapy on patients with advanced small-cell lung cancer while capturing their distinctive characteristics receiving such treatment. Common biomarkers for immunotherapy, such as PD-L1 expression and TMB assays, are costly, time-consuming, and inadequate predictors for ES-SCLC outcomes (Wang et al. 2022; Reck et al. 2019; Antonia et al. 2016). Therefore, it is imperative to identify predictive indicators that are accurate, cost-effective, and easily accessible for early detection of potential benefits associated with immunotherapy. Consequently, this research led to developing a column-line graphical representation capable of predicting survival rates among patients with ES-SCLC undergoing immune checkpoint inhibitor treatments by incorporating clinicopathological traits along with selected inflammatory markers.

Due to the aggressive nature of SCLC and the high risk of early-stage distant metastases, some patients present with advanced disease at the time of diagnosis. Liver, bone, and brain are common sites for metastasis. Several significant clinical studies, including the IMpower133 study (Horn et al. 2018) and the CAPSTONE-1 study (Wang et al. 2022), have reported improved survival rates in ES-SCLC patients without liver metastases when treated with immune-combination chemotherapy. Moreover, a retrospective analysis involving 236 ES-SCLC patients (Xie et al. 2023) identified liver metastasis as an independent prognostic factor for survival in ES-SCLC patients who received initial chemotherapy with ICIs (HR = 2.08; 95% CI 1.3–3.82; *P* = 0.018). Similarly, our study demonstrated that ES-SCLC patients had a poor prognosis if they had pre-existing liver metastases before initiating immune combination treatment, which acted as an independent risk factor (HR = 2.59; 95% CI 1.45–4.60; *P* = 0.001). It is plausible that liver metastases impair the body’s anti-tumor defense mechanism by reducing circulating CD8 ^+^ T cells following their interaction with macrophages. This observed immunosuppressive state may originate from liver metastases and might not be alleviated by ICI therapies (Tsilimigras et al. 2021). In addition to liver metastases, our findings suggest a correlation between bone metastases and inferior patient outcomes (HR = 1.99; 95% CI 1.16–3.41; *P* = 0.013). However, the impact of bone metastases on the prognosis of small-cell lung cancer patients receiving immune checkpoint inhibitor therapy remains controversial. Studies (Lee et al. 2022; Xie et al. 2023) have demonstrated that SCLC patients with pre-existing bone metastases who undergo immune-combination therapy experience significantly reduced average survival rates compared to those without such metastases (*P* < 0.05). An increase in bone-related events associated with bone metastases, including pain, hypercalcemia, pathological fractures and spinal cord compression disorders may significantly affect patient quality of life and alter immunotherapy outcomes. Nevertheless, studies(Xie et al. 2023) have shown that isolated bone metastasis does not predict overall survival in ES-SCLC patients undergoing initial chemotherapy with ICIs (HR = 1.15; 95% CI 0.71–1.88; *P* = 0.572), suggesting that other factors may obscure the predictive significance of bone metastasis in ICI therapy and warrant further investigation.

Studies have indicated a correlation between NLR, which often reflects the inflammatory and immune status of the body, and outcomes in patients undergoing immunotherapy for various cancers (Russo et al. 2020; Fucà et al. 2019). However, the association between NLR and prognosis in SCLC remains controversial. In a retrospective study (Liu et al. 2023a) involving 612 SCLC patients receiving initial chemotherapy (with 22 also receiving PD-L1 inhibitors), NLR emerged as an independent predictive factor for SCLC cases (*P* = 0.004). Moreover, our findings suggest that patients with SCLC and an NLR ≥ 2.14 generally exhibit improved post-ICI treatment prognosis (HR = 0.42; 95% CI 0.23–0.77; *P* = 0.005). Additionally, two other investigations (Chen et al. 2021; Xie et al. 2015) focusing on SCLC patients reported similar results. However, further analysis of 53 ES-SCLC patients treated with atezolizumab and chemotherapy in the NCT03041311 trial (Zhao et al. 2023) demonstrated that NLR was not a reliable predictor for ES-SCLC patients (*P* = 0.69). Furthermore, a retrospective study (He et al. 2021) involving 234 SCLC patients concluded that pre-treatment NLR with platinum-based chemotherapy alone did not independently predict overall survival in ES-SCLC patients. In conclusion, the relationship between NLR and prognosis in SCLC remains subject to debate and necessitates further investigation for validation and comprehension purposes. Consequently, additional research is imperative to comprehensively evaluate the predictive value of NLR in SCLC patients to provide more accurate and dependable guidance for clinical prognostic assessments.

LDH, which plays a pivotal role in the conversion of pyruvate to lactate, is crucial for glycolysis in cancerous cells and serves as a frequent marker of tumor cell activity (Liu et al. 2023a; Sun et al. 2022). Our study findings suggest that patients with ES-SCLC who have an initial LDH level of 146.5 U/L or higher tend to exhibit a diminished response to immunotherapy (*P* = 0.037). However, the precise mechanism linking LDH and immunotherapy remains unclear; hypotheses propose that elevated LDH levels leading to lactic acid production impair the ability of T cells and NK cells to generate NFAT in acidic environments, resulting in reduced interferon gamma release and weakened lymphocyte-mediated tumor cell destruction (Zhang et al. 2019). Moreover, multiple studies indicate that LDH acts as an unfavorable prognostic factor for SCLC patients by potentially predicting brain and bone metastasis (Anami et al. 2019; Conen et al. 2016). Therefore, integrating serum LDH measurements with additional metrics is essential for assessing risk stratification and future prospects in ES-SCLC.

Several studies have identified the combination of LDH and the dNLR, known as LIPI, as an independent prognostic factor in SCLC (Sun et al. 2022; Yang et al. 2022). Based on these prognostic factors, LIPI is categorized into three groups: good (0 factors), medium (1 factor), and poor (2 factors). The prognostic factors include a dNLR ≥ 3 and an LDH level ≥ the upper limit of normal. This study found that among patients with ES-SCLC receiving combination immunotherapy, those with a poor LIPI score had a significantly increased risk of adverse prognosis compared to patients with a good LIPI score (HR = 8.79; 95% CI 3.05–25.29; *P* < 0.001). Recently, Chinese scholars conducted a large multicenter retrospective analysis focusing on SCLC patients receiving first-line atezolizumab/durvalumab combined with standard chemotherapy (Zhao et al. 2023). The results also demonstrated a significant correlation between LIPI and survival prognosis (*P* < 0.05). LIPI has consistently shown strong immunopredictive power for SCLC across multiple studies. It possesses advantages such as minimal invasiveness and ease of acquisition, making it poised to become a widely used clinical predictive tool for SCLC immunotherapy in the future.

Albumin, a well-known immunogenic protein, is often associated with malnutrition and widespread inflammation due to its low levels. Numerous studies have shown that albumin plays an inhibitory role in the systemic inflammatory response and may contribute to tumor progression (Kawata et al. 2021). The PNI, which combines albumin and lymphocytes, is a crucial metric that accurately reflects the correlation between a patient’s nutritional status and overall inflammatory response. A previous study involving 112 men with stage III SCLC found an association between lower predictive nutritional indices and adverse outcomes in these individuals (*P* = 0.009) (Yang et al. 2023). Furthermore, another investigation of patients with limited-stage small cell lung cancer revealed that those with higher PNI values often had more favorable prognoses (*P* = 0.018) (Qiu et al. 2022). The cut-off value of PNI in this study was determined to be 51.03, consistent with previous studies (45.15–52.525) (Yang et al. 2023; Qiu et al. 2022; Wang et al. 2020). Our findings demonstrate that individuals with a PNI exceeding 51.03 generally exhibit improved outcomes (HR = 0.36; 95% CI 0.19–0.69; *P* = 0.002), corroborating prior research suggesting that ES-SCLC patients with adequate nutrition are more likely to benefit from immunotherapy effectively. Additionally, malnutrition is notably prevalent in various cancers including lung cancer (Marshall et al. 2019), characterized by intricate interactions among malnutrition, tumor proliferation, metastasis leading to further malnutrition development within patients while simultaneously exacerbating tumor spread—forming an unyielding cycle. The present study indicates potential associations of these mechanisms with weakened immune responses, inflammatory reactions, leptin concentrations as well as tumor cell autophagy among cancer patients (Dias Rodrigues et al. 2017; Inagaki-Ohara 2019; Görgülü et al. 2020).

In conclusion, the presence of liver and bone metastases, NLR below 2.14, a reduced LIPI score, PNI less than 51.03, and LDH levels exceeding 146.5 collectively indicate an unfavorable prognosis for individuals with ES-SCLC prior to receiving immunological combination treatment. These factors were utilized to develop a predictive model. By analyzing Nomogram plot, patient risk scores were determined, demonstrating improved post-immunotherapy outcomes and higher survival probabilities at 12, 18, and 24 months for those with lower scores. The predictive model achieved a Harrell’s C-index of 0.84 (95% CI 0.75–0.92) during training and 0.88 (95% CI 0.76–0.99) in the validation set, respectively, indicating its efficacy in stratifying patients’ prognoses. Survival forecasts at 12, 18, and 24 months yielded AUC values of 0.778, 0.908, and 0.845 in the training phase and 0.808, 0.844, and 0.824 in the validation phase, respectively, demonstrating the discriminative power of the model for prognosis prediction across different time points post-immunotherapy among patients with ES-SCLC.

In previous studies, researchers have endeavored to develop innovative predictive models for the survival rates of patients with SCLC. In a multicenter retrospective analysis (Song et al. 2023). The researchers included a total of 2309 SCLC patients from Shandong Province, China, by analyzing patient characteristics and constructing a prognostic model. The results demonstrated that the tAUC value of this model for predicting 1-, 3-, and 5-year OS in both training and validation cohorts were found to be 0.699/0.683/0.683 (training) and 0.698/0.698/0.683 (validation), respectively. Despite its ability to incorporate a substantial sample size enhancing result authenticity and reliability, this approach has limitations such as excluding certain inflammatory markers and other laboratory indicators while overlooking aspects related to immunotherapy methods. In a subsequent retrospective study (Liu et al. 2023a), a cohort of 612 patients diagnosed with SCLC was analyzed to develop a predictive model for patient outcomes. Harrell’s C index for both the training and validation datasets of this model were found to be 0.666 and 0.747 respectively. The strength of this model lies in its comprehensive inclusion of diverse samples and inflammatory markers, enabling an in-depth analysis of patient outcomes. Additionally, it is worth noting that the model does not account for factors like specific types of immunotherapy utilized which may potentially impact its predictive capability.

In contrast to the aforementioned models, our method primarily aims to predict the progression of ES-SCLC in patients undergoing immune-combination therapy, thereby enhancing its applicability in current clinical settings. Moreover, it incorporated NLR, LIPI, LDH, PNI, liver metastasis and bone metastasis, which reflect the inflammatory and nutritional status of patients and correlate them with prognosis. Notably, both Harrell’s C index and our model’s survival predictions demonstrate higher AUC values at different time points in both the training and validation datasets compared to the previously mentioned models; this indicating superior accuracy and discrimination capabilities of our model. However, it is important to acknowledge that our model has limitations due to its relatively small sample size. Despite this limitation though smaller than those used in previous studies—our model provides precise and valuable prognostic forecasts. Overall, this approach offers distinct advantages in predicting survival probability among patients with ES-SCLC following immunological combination therapy.

At the 2022 World Conference on Lung Cancer (WCLC), Johal et al. (2022) investigated the association between responder health and prognosis in participants who received initial durvalumab and chemotherapy during milestone time points (weeks 6, 12, and 20) of the CASPIAN study. Their findings revealed a significant enhancement in both PFS and OS among responders compared to non-responders (all P < 0.001). Notably, by week 12, responders exhibited a mOS of 16.7 months (14.4–21.5), contrasting with non-responders’ mOS of 8.0 months (5.7–8.7). Responders also demonstrated a prolonged mPFS of 5.7 months (5.0–7.0), while non-responders had an mPFS of only 1.8 months (1.7–1.8). The research highlighted a substantial correlation between response status at milestone time points and prognosis in SCLC patients undergoing initial durvalumab combination chemotherapy, where responders displayed significantly improved PFS and OS compared to non-responders.

Based on these findings, this study further investigated the association between response levels and prognosis of ES-SCLC at milestone real-world time points. The observations revealed a significant improvement in OS among individuals who exhibited a response to immune checkpoint inhibitors at weeks 6, 12, and 20 compared to non-responders. Notably, responders demonstrated a greater extension in mPFS than non-responders at weeks 6 and 20. By the end of week 12, there was a trend towards an extended mPFS; however, no statistically significant difference in PFS was observed between responders and non-responders (mPFS: 9.2 months vs. 6.3 months, *P* = 0.069). Our study findings indicate that during milestone time phases of administering immune checkpoint inhibitors for ES-SCLC treatment, responders exhibit substantial improvements in both OS and PFS rates compared to non-responders. In contrast to the preliminary results from the CASPIAN study, our study did not find any significant statistical differences in PFS between responders and non-responders only at the end of week 12; however, during other crucial time periods, both OS and PFS durations were significantly longer in responders than in non-responders. This implies that in real-world scenarios, the treatment response during milestone time points can accurately predict immunotherapy outcomes in patients with ES-SCLC.

The remarkable efficacy of immune checkpoint inhibitors in the management of ES-SCLC has rekindled patients’ hopes for prolonged survival. Currently, several researchers (Liu et al. 2023b; Plaja et al. 2021; Maneenil et al. 2017) are investigating the correlation between clinical symptoms and extended survival in ES-SCLC patients. A study conducted by Liu et al. (2023b) demonstrated that ES-SCLC patients who received initial treatment with atezolizumab and chemotherapy, characterized by an ECOG score of 0 (OR = 1.8, *P* = 0.03), LDH ≤ ULN (OR = 1.8, *P* = 0.03), and a reduced number of metastatic sites (OR = 0.8, *P* = 0.03), exhibited an increased likelihood of sustained LTS. Furthermore, our study evaluated the association between clinical characteristics of these patients and LTS, defining LTS as individuals who survived for 12 months or more following ICIs therapy. Research findings have indicated that individuals without liver or bone metastases prior to combination immunotherapy displayed similar outcomes in terms of OS compared to those observed in the IMpower133 study (Horn et al. 2018) and CAPSTONE-1 study (Wang et al. 2022), suggesting a potential benefit for improved OS among patients without liver metastases treated with atezolizumab or adebrelimab alongside chemotherapy (HR = 0.64, 95% CI 0.45–0.90; HR = 0.61, 95% CI 0.46–0.81).

The limitations of our study primarily stem from its retrospective design, which hinders the ability to address potential biases and confounding factors, such as recall bias in patients previously treated with immune checkpoint inhibitors. Moreover, the small sample size significantly limits the generalizability of our findings. Additionally, we did not consider the heterogeneity of immune checkpoint inhibitor recipients, which may impact prognosis outcomes. Lastly, our monitoring period was limited to 12 months compared to previous studies reporting long-term survival ranging from 18 months to 5 years. Therefore, future comprehensive and prospective multi-institutional studies are essential for external validation of our model and exploration of its clinical significance for SCLC patients undergoing immunotherapy at different cancer centers. These investigations aim to comprehensively evaluate prognostic factors and identify personalized treatment strategies.

## Conclusion

This study developed a prognostic model for patients with ES-SCLC undergoing immunotherapy, incorporating six indicators including liver metastasis, bone metastasis, NLR, LIPI, PNI, and LDH based on patients’ clinical characteristics and hematological markers. The model demonstrates excellent discriminative ability and calibration, enabling the prediction of survival probability in ES-SCLC patients receiving immunotherapy.

In the real world, the response status at milestone time points (6, 12, and 20 weeks) can serve as a good indicator of long-term survival for patients with ES-SCLC receiving immunotherapy. This allows for early survival predictions during the treatment process, providing a reference for clinical screening of the population that benefits from immunotherapy.

## Data Availability

More detailed data are available from the corresponding authors upon reasonable request.
